# Transient Post-Natal Exposure to Xenoestrogens Induces Long-Term Alterations in Cardiac Calcium Signaling

**DOI:** 10.3390/toxics10030102

**Published:** 2022-02-23

**Authors:** Cassandra Tabasso, Marie-Pauline Frossard, Camille Ducret, Hassib Chehade, Claire Mauduit, Mohamed Benahmed, Umberto Simeoni, Benazir Siddeek

**Affiliations:** 1 Woman-Mother-Child Department, Division of Pediatrics, Developmental Origins of Health and Disease (DOHaD) Laboratory, Centre Hospitalier Universitaire Vaudois and University of Lausanne, 1011 Lausanne, Switzerland; cassandra.tabasso@unil.ch (C.T.); marie-pauline.frossard@unil.ch (M.-P.F.); camille.ducret@eduvaud.ch (C.D.); hassib.chehade@chuv.ch (H.C.); umberto.simeoni@chuv.ch (U.S.); 2 Institut National de la Santé et de la Recherche Médicale (INSERM) U1065, Centre Méditerranéen de Médecine Moléculaire (C3M), Team 10, 06204 Nice, France; claire.mauduit@univ-lyon1.fr (C.M.); mohamed.benahmed@unice.fr (M.B.)

**Keywords:** endocrine disruptor, post-natal, heart, calcium signaling, programming

## Abstract

Today, non-communicable disorders are widespread worldwide. Among them, cardiovascular diseases represent the main cause of death. At the origin of these diseases, exposure to challenges during developmental windows of vulnerability (peri-conception, in utero, and early infancy periods) have been incriminated. Among the challenges that have been described, endocrine disruptors are of high concern because of their omnipresence in the environment. Worrisomely, since birth, children are exposed to a significant number of endocrine disruptors. However, the role of such early exposure on long-term cardiac health is poorly described. In this context, based on a model of rats exposed postnatally and transiently to an estrogenic compound prototype (estradiol benzoate, EB), we aimed to delineate the effects on the adult heart of such transient early exposure to endocrine disruptors and identify the underlying mechanisms involved in the potential pathogenesis. We found that this transient post-natal exposure to EB induced cardiac hypertrophy in adulthood, with increased cardiomyocyte size. The evaluation of cardiac calcium signaling, through immunoblot approaches, highlighted decreased expression of the sarcoplasmic reticulum calcium ATPase 2 (SERCA2) and decreased Nuclear Factor of Activated T Cells (NFAT3) phosphorylation as a potential underlying mechanism of cardiac hypertrophy. Furthermore, the treatment of cardiomyocytes with EB in vitro induced a decrease in SERCA2 protein levels. Overall, our study demonstrates that early transient exposure to EB induces permanent cardiac alterations. Together, our data highlight SERCA2 down-regulation as a potential mechanism involved in the cardiac pathogenesis induced by EB. These results suggest programming of adult heart dysfunctions such as arrhythmia and heart failures by early exposure to endocrine disruptors and could open new perspectives for treatment and prevention.

## 1. Introduction

Non-communicable diseases (NCDs) are a growing concern in the world. They include diabetes, infertility, cancers, neuropathology, and cardiovascular diseases. Together, NCDs cause 71% of the total deaths in the world. Among them, 43% are represented by cardiovascular diseases (World Health Organization, 2018). In NCDs’ etiology, evidence points to the determinant role of the fetal and neonatal periods. As such, the DOHaD (Developmental Origins of Health and Disease) hypothesis explains how the organism faces windows of vulnerability to its external environment, first the maternal and then the external environment, when its development is still extremely plastic [1,2,3,4]. During these windows of vulnerability, the experience of certain challenges such as stress, nutrition (e.g., high-fat diet), or factors from the environment (such as pollutants) represents important risk factors for NCDs in adulthood. Among them, endocrine disruptors are of particular concern [5,6]. Natural or synthetic endocrine disruptors are omnipresent in everyday life, and once inside the body, they disrupt the endogenous hormone signaling. Most endocrine-disrupting chemicals (EDCs) act as xenoestrogens. Worrisomely, baby- and children-oriented products such as diapers, bottles, toys, and cosmetics contain a significant amount of EDCs. To this day, the impact of early developmental exposure to EDCs has been studied mostly on hormone-dependent organs such as the reproductive system or, more recently, also on the central nervous system; however, very little is known of their impact on the heart [7]. Indeed, only a few studies described the impact of EDCs on the heart. Among them, one study showed that acute exposure to bisphenol A (BPA), used widely in plastic or epoxy resins, caused arrhythmia and heart failure [8,9]. Phthalates, which are common plasticizers, were shown to impair contractility as well as heart rate [7]. The promotion of arrhythmia by estrogenic compounds has been explained by their potential to regulate myocyte Ca^2^⁺ handling, where they increase sarcoplasmic reticulum Ca^2^⁺ leak [9]. In the regulation of heart rate and contractility, the process of “excitation-contraction coupling” (ECC) describes how a potential action induces the release of Ca^2+^ from intracellular stores, which initiates cardiac contraction. Ca^2+^ is a crucial mediator in heart function [10]. Pacemaker cells depolarize the membrane, which activates the L-type calcium channels and induces Ca^2+^ entry in the cardiomyocyte [11]. The rise of Ca^2+^ concentration in the cytoplasm triggers a calcium release through the ryanodine receptor (RyR), a process referred to as calcium-induced calcium release. This Ca^2+^ binds Troponin C, thereby initiating muscle contraction. In order not to lose Ca^2+^, sarcoplasmic reticulum calcium ATPase 2 (SERCA2) pumps it actively back into the sarcoplasmic reticulum. The Ca^2+^ concentration decreases, and the contraction eventually stops. Contraction frequency can be regulated by the sympathetic nervous system (SNS) through phosphorylation–dephosphorylation cascades. When the SNS is triggered and starts releasing neurotransmitters, the β1 adrenergic receptor causes activation of the G protein and subsequent rise of the cAMP levels, activating consequently protein kinase A (PKA C), which phosphorylates the L-type calcium channel and RYR, thus increasing Ca^2+^ entry in the cytoplasm. At the same time, it also phosphorylates phospholamban, stopping the inhibition of SERCA2 and increasing Ca^2+^ recovery in the sarcoplasmic reticulum. These activations are continued as long as the neurotransmitter (noradrenaline) is produced, therefore causing an increased heart rate. In the context of exposure to EDCs, while previous investigations on the heart highlighted their impact on calcium signaling and arrhythmia, the investigations were focused mostly on acute exposures, and covered the exposure of adult animals or in vitro models. Therefore, the long-term impact of EDCs on the heart following early exposure during developmental windows of vulnerability is mostly unknown. In this context, we aimed to define the impact of transient postnatal exposure to an EDC with estrogenic activity on long-term cardiac health and delineate the role of calcium signaling in the potential cardiac alterations.

## 2. Materials and Methods

### 2.1. Materials

The H9C2 cell line, estradiol benzoate, the polyvinylidene difluoride (PVDF) membrane, the RIPA buffer and the anti-β-ACTIN antibody were acquired at Merck KGaA (Darmstadt, Germany). Antibodies raised against A phospho-Phospholamban (Ser16/Thr17), Phospholamban, phospho-PKA C (Thr197), PKA C-α, and SERCA2 were bought from Cell Signaling Technology (Danvers, MA, USA). The ProLong Gold Antifade Reagent with DAPI, Anti-rabbit and anti-mouse HRP, western atto plus chemiluminescent substrate, BCA Protein Assay, D-PBS, DMEM, RIPA buffer, High-Capacity Reverse Transcription Kit, Sybergreen master mix, primers, FITC anti-mouse-conjugated secondary antibody, anti-8-OHdG, and wheat germ agglutinin Texas Red™-X Conjugate were bought from ThermoFisher Scientific (Waltham, MA, USA). Clarity Western ECL Substrate was bought from BioRad (Hercules, CA, USA). The RNA isolation kit was bought from Qiagen GmbH (Hilden, Germany). Bovine Serum Albumin, Acrylamide, and the Cosy pre-stained protein ladder were purchased from Axonlab AG (Dättwil AG, Switzerland). The antibodies raised against NFAT3 and phospho-NFAT3 were bought from Lucerna-Chem AG (Luzern, Switzerland).

### 2.2. Animal Experiment

The experiments with animals were performed in accordance with EU legislation (Directive 2010/63/EU) and approved on 14 January 2013 by a local animal care and use committee (agreement number: NCE-2013-109). Eighteen Male Sprague Dawley rats, from five different litters, were subcutaneously injected with an estrogen-like compound (estradiol benzoate, EB, diluted in corn oil) at a dose of 2.5 μg/day, during the 5 first days of life. Eighteen control animals, from five different litters, received corn oil (vehicle). The dose was selected based on previous experiments [12,13]. All animals were weighed before euthanasia, at post-natal day 77. Euthanasia was performed by CO_2_ inhalation. Hearts were fixed in 4% paraformaldehyde and embedded in paraffin for histological analyses or snap frozen in liquid nitrogen and stored at −80 °C for molecular analyses.

### 2.3. Histological Analyses

Heart sections (5 μm) were stained with Masson’s trichrome or Hematoxylin-Eosin. Image acquisition was performed on an Eclipse Ti microscope (Nikon Europe BV, Amsterdam, Netherlands). Evaluation of the extracellular matrix depot was performed as described by Chen Y et al. [14] with Image J software.

### 2.4. Western Blots Analysis

Proteins were extracted from frozen cardiac tissue powder or from H9C2 cells with RIPA buffer. First, 20 to 40 µg of the samples were separated on SDS-PAGE as previously described [15]. After the transfer, the membranes were blocked with PBST-BSA (3% Albumin). Primary antibodies for PLN, P-PLN, PKA C alpha, and SERCA 2 were used (diluted at 1/1000 in PBST-BSA 1%). Secondary antibodies (anti-mouse or anti-rabbit-HRP, according to the primary antibodies, diluted 1/5000 in PBST-BSA 1%) were added, and the proteins were revealed with ECL. In order to check for the equal loading of the proteins, the membranes were re-incubated with anti-GAPDH (diluted 1/4000 in PBST-BSA 1%). A luminescent image analyzer camera G: Box (Syngene, Cambridge, UK) was used for luminescent signal scanning. The signals were quantified with Gene Tools software (Syngene, Cambridge, UK).

### 2.5. RNA Isolation

Total RNA was isolated from frozen heart powder using the miRneasy kit according to the manufacturer’s protocol. The RNA purity was checked via optical density measurement at 230, 260, and 280 nm. The total RNA quantity was evaluated with a NanoDrop Microvolume Spectrophotometer (ThermoFisher Scientific, Waltham, MA, USA). The RNA integrity was evaluated on an agarose gel.

### 2.6. Real-Time Quantitative PCR

In total, up to 500 ng of RNA was reverse transcribed using the High-Capacity Reverse Transcription Kit according to the manufacturer’s protocols and as described previously [16]. The primers’ sequences were *Serca2* forward: GGCTCGTGGGCTCCATCTGC; *Serca2* reverse: TCCAGTATTGCAGGCTCCAGGT; *Gapdh* forward: AAAGCTGTGGCGTGATGG; *Gapdh* reverse: TTCAGCTCTGGGATGACCTT. Data were normalized to *Gapdh* using the ΔCT method.

### 2.7. Cell Treatment

H9C2 cardiomyocyte cell lines were cultured in 6-well plates in DMEM with high glucose and 10% FBS with penicillin and streptomycin, in an incubator at 37 °C and 5% CO2. Each day, the medium in the wells was replaced with a medium containing EB (10 nM, 100 nM, and 1000 nM dissolved in ethanol) or 0.001% ethanol (EB0), for 72 h.

### 2.8. Immunofluorescence

To evaluate the cardiomyocyte’s surface, heart sections (5 μm) were stained with wheat germ agglutinin according to the manufacturer’s protocol, Masson’s trichrome, or Hematoxylin-Eosin. Image acquisition was performed on a Nikon Eclipse Ti microscope (Nikon Europe BV, Amsterdam, Netherlands). Evaluation of the extracellular matrix depot was performed as described by Chen Y et al. [14] with Image J software. To evaluate cellular damage related to oxidative stress, H9C2 cells were grown on coverslips, treated with EB or ethanol (vehicle) for 72 h, and stained with specific antibodies. Cells were washed with D-PBS and fixated with 4% paraformaldehyde. Cells were then permeabilized in PBS-triton (0.1%) and blocked with PBS-BSA 5%. Cells were incubated with primary antibodies, anti-8-OHdG (1/200 in PBS-BSA 1%), and secondary antibodies bound to FITC (1/200 in PBS-BSA 1%). The glass coverslips were then mounted on slides, using a mounting medium containing DAPI. Cells were observed on a Nikon Eclipse Ti microscope. The fluorescence signal corresponding to 8-OHdG was evaluated with ImageJ software.

### 2.9. Data Analysis

The data from the different experiments were analyzed with GraphPad Prism software version 9.1.0 (GraphPad Software, LLC, San Diego, CA, USA.). The values were expressed as the mean ± SEM to account for sample and animal variation within a dataset. Student’s *t*-test was performed to determine whether there were differences between the two groups and an analysis of variance (ANOVA) with Fisher’s LSD test when multiple groups were compared. *p*  <  0.05 was considered statistically significant.

## 3. Results

### 3.1. Transient Postnatal Exposure to EB Induces Cardiac Hypertrophy at Adulthood

**Figure 1:**
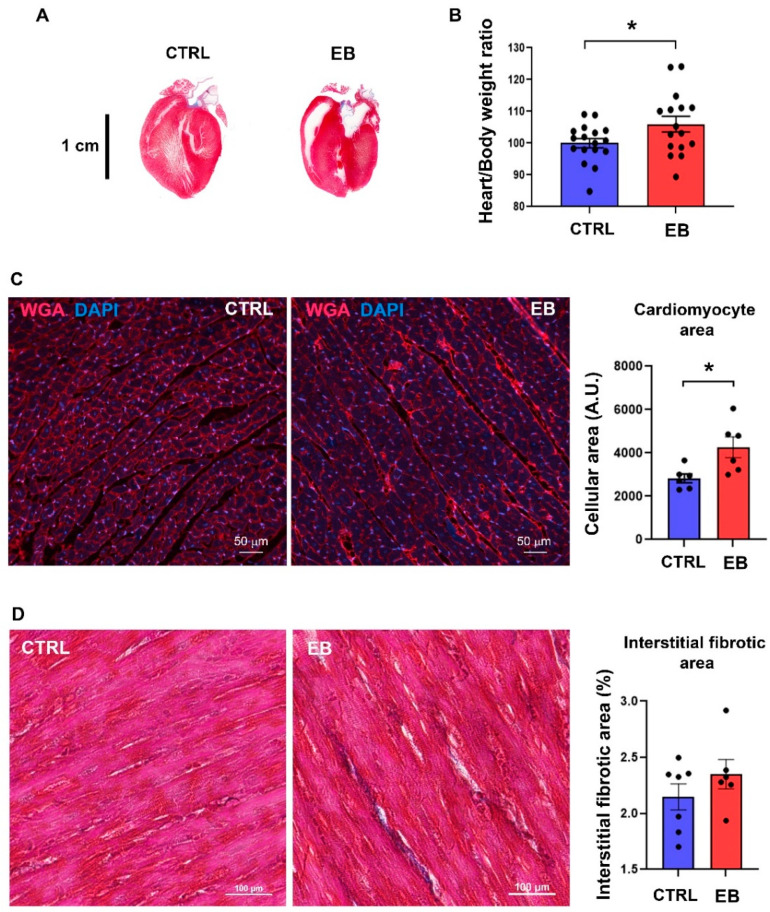
Effects of transient post-natal exposure to estradiol benzoate on adult heart. (**A**) Longitudinal sections of whole heart stained with Masson’s trichrome, from adult rats exposed to corn oil (vehicle, CTRL) or estradiol benzoate (EB) (scale bar, 1 cm). (**B**) Heart versus body weight ratio of animals exposed to corn oil (vehicle, CTRL) or estradiol benzoate (EB). Data are expressed as mean ± SEM; *n* = 17–18 per group. Heart sections from 6 rats exposed to corn oil (CTRL) or estradiol benzoate (EB) were stained with wheat germ agglutinin (WGA) to evaluate cell surface (scale bar, 50 μm) (**C**) and Masson’s trichrome to evaluate extracellular matrix depot (scale bar, 100 μm) (**D**). Student’s *t*-test was performed to compare control and EB-treated animals. * indicates statistical significance (*p* < 0.05).

Hearts were weighed and histological analysis was conducted (Figure 1A,B). At post-natal day 77 (PND77), while no difference was detected in the body weight of animals exposed to corn oil (vehicle, CTRL) compared to rats exposed to estradiol benzoate (EB), an increase in heart relative weight was observed in animals exposed to EB (Figure 1B). After wheat germ agglutinin (WGA) staining, the measurement of cell surface on cross-sectioned cardiomyocytes indicated an increase in cell size in adult rats exposed to EB (Figure 1C). No modification in the extracellular matrix depot was observed by Masson’s trichrome staining (Figure 1D). These data indicate that transient postnatal exposure to EB induces long-term cardiac alterations with hypertrophic patterns.

### 3.2. Early Exposure to EB Induces Long-Term SERCA 2 Downregulation in the Heart

**Figure 2:**
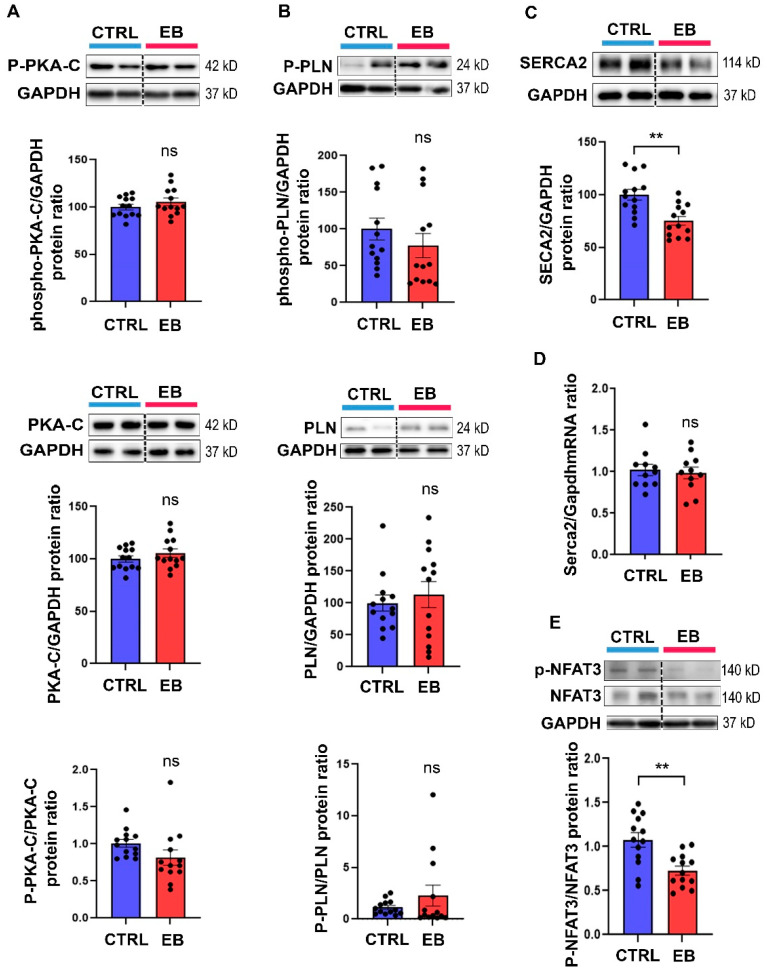
Post-natal exposure to estradiol benzoate alters calcium signaling in the adult heart. Protein levels of (**A**) phospho-PKA C-αPKA C-α, (**B**) phospho-PLN, PLN, and (**C**) SERCA2 protein levels were analyzed at post-natal day 77 in the heart of male rats exposed to corn oil (CTRL) or estradiol benzoate (EB) via Western blot. GAPDH protein levels were used as loading control. Data are expressed as mean ± SEM; *n* = 13 per group. (**D**) *Serca2* mRNA levels were measured by RT-qPCR in the heart of CTRL and EB-treated rats at post-natal day 77. Data are expressed as mean ± SEM; *n* = 11 per group. (**E**) NFAT3 (phospho Ser289) and total NFAT3 protein levels were analyzed at post-natal day 77 in the heart of male rats exposed to corn oil (CTRL) or estradiol benzoate via Western blot. GAPDH protein levels were used as loading control. Data are expressed as mean ± SEM; *n* = 13 per group. Student’s *t*-test was performed to compare control and EB-treated animals. ns: Not statistically significant, ** *p*  <  0.01.

To identify cellular mechanisms potentially involved in these tissue cardiac alterations, we investigated cardiac calcium signaling. Indeed, in other models of exposure to endocrine disruptors, a Ca^2+^ leak was reported [9]. We thus measured the expression levels of phosphorylated protein kinase A alpha (P-PKA C-α) (Figure 2A), total PKA C-α (Figure 2A), phosphorylated phospholamban (P-PLN) (Figure 2B), total PLN (Figure 2B), and sarcoplasmic reticulum calcium ATPase 2 (SERCA2) (Figure 2C) at the protein level via Western blot analyses. We did not detect any difference in the total protein and phosphorylated protein levels of PKA C-α and PLN (Figure 2A,B) between animals exposed to EB and the control animals. In contrast, SERCA2 protein levels were significantly downregulated in the hearts of EB-treated animals (Figure 2C). To determine whether the SERCA2 decrease involved transcriptional regulation, we measured mRNA levels via RT-qPCR and observed no difference between the control and the EB-treated animals (Figure 2D). These data suggest that SERCA2 down-regulation induced by EB involved post-transcriptional mechanisms of regulation.

### 3.3. Early Exposure to EB Induces Decreased NFAT3 Phosphorylation Levels in the Heart

Because a SERCA2 decrease has been shown to induce cardiomyocyte hypertrophy through the induction of Nuclear Factor Activated T Cells (NFAT) de-phosphorylation and activation, we evaluated NFAT3 phosphorylation levels in the hearts of our animals. In the EB-treated group, consistent with SERCA2 downregulation, we observed a significant decrease in NFAT3 phosphorylation levels compared to the control group (Figure 2E).

### 3.4. EB Induces SERCA2 Decrease in Cardiomyocytes In Vitro

**Figure 3:**
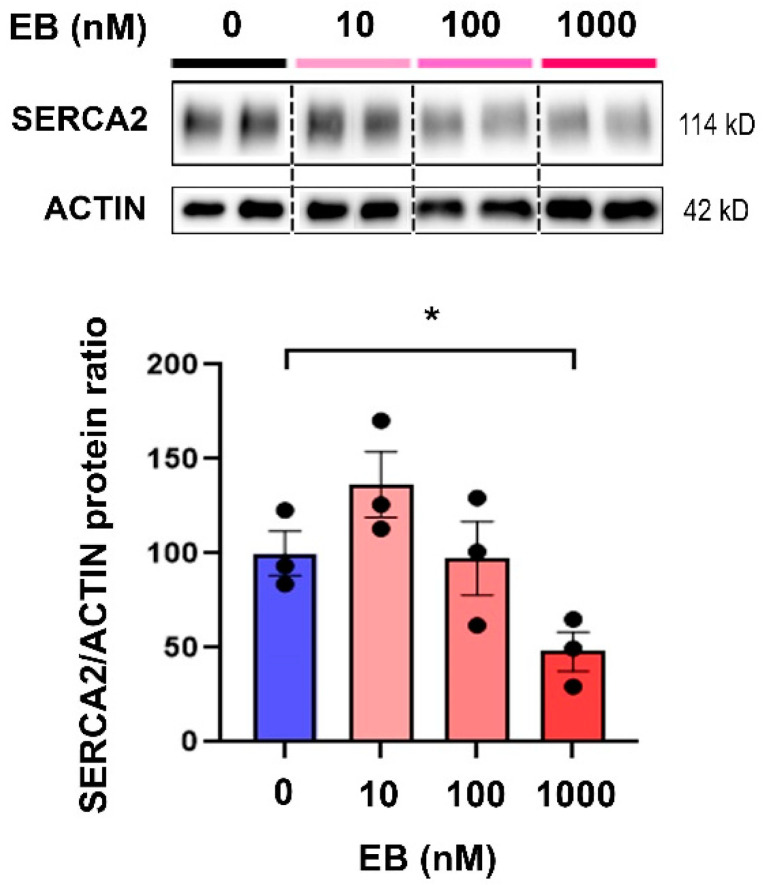
Estradiol benzoate influences SERCA2 expression in cardiomyocytes. H9C2 cells plated in 6-well plates were treated for 72 h with estradiol benzoate at 10 nM, 100 nM, and 1000 nM, or ethanol (EB0). In these cells, SERCA2 protein levels were measured via Western blot. β-ACTIN was used as loading control. Data are expressed as mean ± SEM; *n* = 3 per group. ANOVA with a Fisher’s LSD test was performed to compare control and EB-treated cells. * *p*  <  0.05. The results are representative of two independent experiments.

We next wondered if the observed EB effects on SERCA2 cardiac levels could be the result of its effects on cardiomyocytes. To verify this hypothesis, we treated H2C9 cardiomyocytes with EB at different doses (10 nM, 100 nM, or 1000 nM) for 72 h. The control cells were treated with the same amount of ethanol (EB0). In these cells, similar to the hearts of our animals, EB (1000 nM) treatment induced a significant decrease in SERCA2 protein levels (Figure 3).

### 3.5. SERCA2 Decrease Is Not Associated with Oxidative Stress in Cardiomyocytes

**Figure 4:**
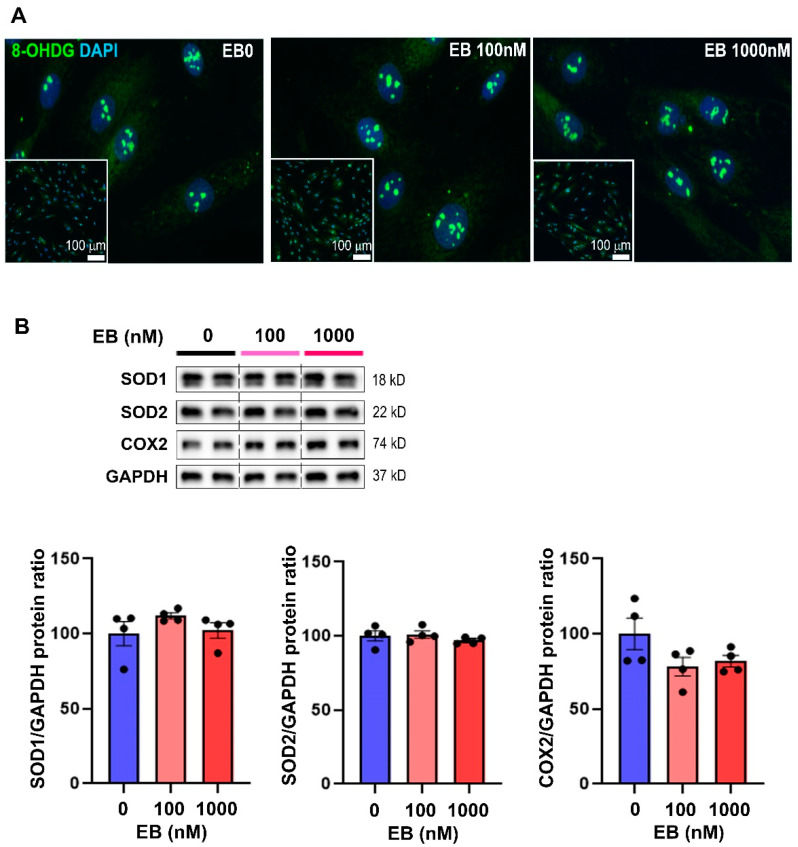
Effects of estradiol benzoate on oxidative stress in cardiomyocytes. H9C2 cells grown on coverslips were incubated with estradiol benzoate at 100 nM and 1000 nM or ethanol (EB0) for 72 h. (**A**) Cells grown on coverslips were incubated with an antibody targeting 8-OHdG (green) (Scale bar = 100 μm). (**B**) In these cells, antioxidant enzymes (Superoxide dismutase 1 and 2, SOD1, SOD2) and the regulator of mitochondrial oxidative phosphorylation COX2 were measured via Western blot. GAPDH protein levels were used as loading control. Data are expressed as mean ± SEM; *n* = 4 per group. ANOVA with a Fisher’s LSD test was performed to compare control and EB-treated cells. ns: not statistically significant.

Oxidative stress plays an important role in the promotion of cardiac hypertrophy and has been also described as a mediator of EDC-related health outcomes in numerous organs. In addition, oxidative stress has been reported to regulate SERCA2 [17]. We thus wondered if SERCA2 downregulation in cardiomyocytes driven by EB was a result of increased oxidative stress. To evaluate oxidative stress, we evaluated the cellular damage induced by oxidative stress in EB-treated cells. 8-hydroxy-2′-deoxyguanosine (8-OHdG), a product of oxidative damage to 2′-deoxyguanosine, is described as a marker for assessing oxidative DNA damage. We thus cultured H9C2 cells on glass coverslips and treated them with ethanol (EB0) or EB at 100 nM and 1000 nM for 72 h, and submitted them to 8-OHdG immunofluorescence staining (Figure 4A). No modification was detected between treated (100 nM and 1000 nM) and untreated cells (EB0). Moreover, in these cells, we measured the expression level of proteins involved in oxidative stress regulation: Superoxy dismutases (SOD) 1 and 2 and cyclooxygenase 2 (COX-2). Using Western blot approaches, we did not detect any change in protein levels between untreated cells and cells treated with EB (Figure 4B). Overall, these data indicate that SERCA2 decrease in cardiomyocytes incubated with EB is not related to oxidative stress.

## 4. Discussion

Exposure to EDCs is a crucial public health issue nowadays, and almost nothing is known about the impact of early exposure on long-term cardiac health. Among the different avenues of exposure, diapers are of high concern. Indeed, it is estimated that during the first three years of life, an infant uses approximately 4340 diapers, which have been reported to contain a significant number of endocrine disruptors such as phthalates [18]. Therefore, it is crucial to understand how EDCs will influence long-term health, so that the exposure can be, if not prevented, at least reduced and controlled. In this study, we showed that early transient exposure to a compound with estrogenic activities (EB) can induce permanent cardiac alterations. Indeed, in rats, we demonstrated transient post-natal exposure to EB-induced cardiac hypertrophy, associated with a decrease in cardiac SERCA2 levels. EB induced SERCA2 decrease in vitro as well, as seen in cardiomyocyte cell lines showing that early EB exposure can affect cardiomyocytes’ Ca^2+^ signaling. As SERCA2 is the only pump responsible for Ca^2+^ reuptake in the sarcoplasmic reticulum, a decrease in SERCA2 levels can directly modify intracellular Ca^2+^ homeostasis and myocyte contractility [19]. Its role has been shown to be crucial in the maintenance of a regular heart rate, the prevention of cellular damage from Ca^2+^ accumulation, and heart failure [20]. Indeed, defects in Ca^2+^ reuptake are worsened by the fact that a high cytoplasmic Ca^2+^ concentration affects the Ryanodine receptor (RyR) through the Ca^2+^ intake-Ca^2+^ release causing a Ca^2+^ leak from the sarcoplasmic reticulum, amplifying the Ca^2+^ accumulation [21]. In this case, accumulation has been shown to generate asynchronous calcium waves and arrhythmic contractions in a model of animals exposed to a high-fat diet [22]. It would thus be of interest to determine whether SERCA2 deficiency in EB-treated cells would increase the Ca^2+^ concentration in the cytoplasm in comparison to control cells. In this sense, the transcription factor NFAT3, which plays a critical role in Ca^2+^/calcineurin-mediated cardiac hypertrophic signaling, was found to be more dephosphorylated in the hearts of EB-treated animals. Indeed, an increased intracellular calcium concentration promotes calcineurin activation and the subsequent dephosphorylation of NFAT. Dephosphorylated NFAT translocates into the nucleus where it stimulates the expression of pro-hypertrophic genes such as the myosin heavy chain (MHC) and brain natriuretic peptide (BNP) [23]. These points support the hypothesis that in EB-exposed animals, SERCA2 downregulation leads to an increased intracellular calcium concentration, which activates the calcineurin-NFAT pathway and finally results in cardiac hypertrophy. Notably, a decreased SERCA2a expression level or activity has also been reported in physiological conditions such as ageing and pathological conditions such as a failing heart [24]. This decrease was associated with a prolongation of contraction time and impaired myocardial function. Similar to our findings, decreased SERCA2 levels have been incriminated in altered calcium signaling and organ dysfunction following exposure to EDCs in other models. For example, pyrethroid insecticides have been shown to inhibit SERCA2 in rat cortical microsomes [25]. Because dysregulation of neuronal intracellular Ca^2+^ can play a crucial role in brain dysfunction, the SERCA2 decrease represents a potential underlying mechanism of EDCs’ neurotoxic effect. In the testis, SERCA is inhibited by exposure to the plasticizer BPA, which modulates Ca^2+^ from intracellular storage [26] and might cause spermatocytes’ death [27] and Sertoli cell dysfunction [28]. Among the potential origins of the SERCA2 decrease, we evaluated the role of oxidative stress. Based on our data, oxidative stress did not seem to play a role in SERCA2 alteration. Other mechanisms that should be further explored are the epigenetic regulations of SERCA2 expression. Indeed, in the developmental origins of health and disease, epigenetics is described as a key player [29]. Interestingly, studies reported DNA methylation [30] and microRNAs [31,32] as regulators of SERCA2. In our model, alteration of SERCA2 due to DNA methylation appeared less likely since no modification was observed in SERCA2 mRNA levels. However, since microRNAs exert their effects at the post-transcriptional level, they represent an interesting target to explore.

In conclusion, our data indicate that early transient exposure to an endocrine disruptor causes permanent alterations in cardiac calcium signaling. Our project offers new insight into understanding the long-term cardiac pathogenesis induced by early exposure to EDCs, and highlights SERCA2 as a potential therapeutic target. In order to reverse SERCA2 alterations, reducing the PLN inhibition on SERCA2 has been tested. This idea was once considered revolutionary, as in rodent models, adenoviral-delivered interferent RNA against PLN in the heart allowed researchers to cure the heart failure phenotype [33,34]. However, in humans, PLN silencing has been associated with lethal cardiomyopathy. The other option would be the direct targeting of SERCA2 [20,35]. Adenovirus-mediated gene transfer of SERCA2 has been developed in rodents and cellular models. Increasing SERCA2 production in a cardiomyocyte has demonstrated benefits for the neighboring cardiomyocytes, and overexpressing this protein restores the wildtype phenotype. This technique, applied in porcine models through a single coronary injection of Adenoviruses carrying SERCA2 genes, has shown promising results after treatment [20]. A dietary approach could also help in ameliorating the phenotype. For instance, in a model of cardiac pressure overload, the stilbenoid resveratrol, which is widely found in grapes and has antioxidant properties, has been reported to prevent the downregulation of SERCA2 and cardiac hypertrophy [36]. Therefore, identifying and understanding the impact of endocrine disruptors on adult cardiac health after early developmental exposure presents possibilities for new treatments and better prevention.

## Data Availability

The data presented in this study are available on request from the corresponding author.
